# Palliative care education: a nationwide qualitative study of emergency medicine residency program directors in the United Arab Emirates

**DOI:** 10.1186/s12245-024-00643-z

**Published:** 2024-05-23

**Authors:** Thana Harhara, Rasha Buhumaid, Leen Oyoun Alsoud, Halah Ibrahim

**Affiliations:** 1 Department of Medicine, Sheikh Khalifa Medical City, Abu Dhabi, United Arab Emirates; 2 College of Medicine, Mohammed Bin Rashid University of Medicine and Health Sciences, Dubai, United Arab Emirates; 3 Department of Emergency Medicine, Rashid Hospital, Dubai Health, Dubai, United Arab Emirates; 4 Department of Medical Sciences, Khalifa University College of Medicine and Health Sciences, Abu Dhabi, United Arab Emirates

**Keywords:** Emergency medicine, Residency, Graduate medical education, Palliative care, End-of-life care

## Abstract

**Background:**

Emergency medicine (EM) physicians routinely care for patients with serious life-limiting illnesses. Educating EM residents to have general skills and competencies in palliative medicine is a global priority. The purpose of this study was to describe the current status of palliative and end-of-life education in EM residency programs in the United Arab Emirates (UAE) and to identify barriers and opportunities to inculcating palliative care (PC) instruction into EM training in a non-Western setting.

**Methods:**

Using the American College of Emergency Medicine’s milestones for Hospice and Palliative Medicine for Emergency Medicine as a question guide, semi-structured interviews were conducted with program directors of all 7 EM residency programs in the UAE from January through July 2023. Qualitative content analysis was conducted to identify recurring themes.

**Results:**

All program directors agreed that PC knowledge and skills are essential components of training for EM residents but have had variable success in implementing a structured PC curriculum. Six themes emerged, namely the educational curriculum, PC policies and practices, comprehensive PC services, cultural and religious barriers to PC, EM scope of practice, and supporting residents after patient death.

**Conclusion:**

UAE national EM residency curriculum development is evolving with an emphasis on developing a structured PC curriculum. As EM residencies implement policies and programs to improve care for patients and families dealing with serious illness, future studies are needed to assess the impact of these initiatives on patient quality of life and physician well-being.

**Supplementary Information:**

The online version contains supplementary material available at 10.1186/s12245-024-00643-z.

## Background

Emergency medicine (EM) was recognized as a specialty in the United Arab Emirates (UAE) in 2000. In 2007, the country’s first EM residency training program was established [1]. The specialty was initially designed to focus on resuscitation, stabilization, and early management of acute disease processes. Over the past two decades, patient demographics in the country’s emergency departments (ED) have followed global patterns as the UAE experiences population aging and an increased prevalence of cancer and chronic, non-communicable diseases [2]. Currently, EM physicians in the UAE routinely care for patients with end-stage disease.

In many Western countries, palliative care (PC) services have developed in parallel with the aging population. PC is a specialized care approach that employs early detection and symptom relief to improve the quality of life for patients and families dealing with serious illness [3]. Initiating PC services in the ED aligns treatment with patient and family preferences and has been shown to have several benefits [4]. For patients, the early identification of PC needs decreases physical and psychological distress and improves symptom management and quality of life [5]. For family members and caregivers, early referral to PC facilitates shared decision-making and eases bereavement adjustment [6]. For institutions, inpatient PC consultations within 24 h are associated with fewer intensive care admissions, decreased in-hospital mortality, shorter inpatient length of hospitalization, and reduced healthcare expenditures [4, 7].

Studies in the United States (US) and Canada suggest that barriers to providing PC in the ED setting include time pressures, lack of access to medical records, uncertainty about the diagnosis and prognosis, and lack of prior patient-physician relationships [8, 9]. Religious and cultural values and preferences can also influence health communication and care provision, particularly in serious illness [10]. In many collectivist cultures, families believe that talking openly about death can lead to loss of hope and accelerate the dying process [11], though recent studies show that family-patient wishes are often discordant and patients prefer to be informed of their diagnosis and treatment options [12, 13]. Formal education in PC has been identified as a key solution to overcoming the barriers to delivering PC in the ED [14]. Skills gained during residency training impact physicians’ practice throughout their careers [15]. Research shows that ED physicians who are trained to prioritize resuscitative and life-saving care often do not consider PC provision within their scope of practice [16]. Therefore, educating EM residents to have general skills and competencies in palliative medicine is a global priority. In this manuscript, we report the findings from a national study of EM Residency Program Directors (PD) in the UAE. Our objectives were to describe the current status of PC and end-of-life education in the country’s EM residency programs and to identify barriers and opportunities for inculcating PC instruction into EM training.

## Methods

Using the COnsolidated criteria for REporting Qualitative research (COREQ) for collecting and reporting our data [17] and the American College of Emergency Medicine (ACEP)’s milestones for Hospice and Palliative Medicine for Emergency Medicine (HPM-EM) [18] to guide our questionnaire and analysis, we conducted semi-structured interviews with all EM PDs in the UAE between January and July 2023.

### Setting and participants

The UAE is a multi-ethnic and multi-cultural country in the Middle East. Over the past several decades, the country has made substantial investments in healthcare and education, with the development of academic medical centers and international accreditation of many institutions and residency programs [19, 20]. Palliative medicine is a small but growing discipline in the country, with PC facilities and hospices currently in development alongside comprehensive cancer centers in several hospitals. Recent studies of UAE medical schools and internal medicine residency programs reveal limited formal PC education but great interest among medical educators in expanding PC training [19]. To date, there is little systematic information available on PC training or care delivery in EM.

EM residency training in the UAE is structured competency-based clinical education of four years duration. Each residency program is led by a single program director (PD). The PD is a senior physician educator who is responsible for all aspects of the residency program, including curriculum development, policy implementation, and program administration and oversees the teaching, supervision, and assessment of the trainees. We conducted a purposive sampling of PDs of all Arab Board-accredited EM residency programs in the country.

### Interview guide

At the time of this study, there was no consensus on PC or end-of-life competencies for EM residents in the UAE. The conceptual underpinnings for this study were based on the ACEP’s HPM-EM curriculum [18]. Developed by an expert, multidisciplinary panel, this curriculum compiles a list of core palliative and end-of-life care domains in EM, covering topics such as communication skills and ethical considerations, pain management, and interprofessional collaboration [18]. Based on the ACEP’s HPM-EM framework, one of the authors (TH) drafted the initial semi-structured interview guide (Appendix E1). The questions aimed to understand the depth and breadth of palliative and end-of-life care education in the residency program, EM resident clinical exposure to patients with PC needs, and their competence in communicating with and caring for patients and families dealing with serious illness. Questions included basic demographic information about faculty, trainees, and rotations and open-ended questions about the content of PC education, teaching methods, assessments, and any planned curricular or policy changes in PC education. We also sought to identify potential challenges the PDs faced in teaching palliative medicine to EM residents. Five emergency medicine physicians who are involved in resident education in the US (*n* = 2) and the UAE (*n* = 3) piloted the questionnaire for length and clarity. Minor contextual changes were made based on their input. These physicians did not participate in the final interviews. We performed data collection and analysis concurrently and iteratively adjusted the interview guide as new information arose.

### Data collection

All PDs from the seven Arab Board-accredited UAE residency programs were identified through institutional websites or personal contacts. The participants were first informed about the study purpose and protocols via email, and when they agreed to participate, a virtual interview was scheduled in advance so that they could be conducted privately and with minimal disruptions. Based on the concept of information power [20] and the high-quality interviews (conducted by two interviewers with content expertise and experience in qualitative interviewing), we believe that the seven PD interviews were sufficient to answer our research questions. Interviews were conducted between January and July 2023 in English and lasted approximately 30–40 min each. They were audio recorded with participant consent, transcribed verbatim, and checked for accuracy. No additional notes were taken. Participants were not identified by name in the audio recordings or transcriptions and, other than basic demographic information, findings were not linked to individual programs. The study was approved by the Khalifa University Research Ethics Committee [H22-022]. Individual written informed consent was obtained from all participants. Participation was voluntary and no incentives were offered.

### Data analysis

We performed all data management, coding, and analysis manually. Two of the authors (HI, TH) independently completed a line-by-line review of each transcript to generate initial codes. We then conducted thematic content analysis to find recurring concepts that were noteworthy or important to the questions we were trying to answer [21]. Following the process of qualitative data analysis, we engaged in iterative and cyclic constant comparison analysis to group these concepts into themes [22]. Through in-depth conversations with the entire research team, we reached consensus on a coding scheme that was applied to all transcripts. To enhance trustworthiness of the data, an audit trail [23] was maintained through member checks with interested participants and a checklist of data entry and analysis with all authors.

### Team characteristics and reflexivity

Our diverse team consists of clinician educators involved in both undergraduate and postgraduate medical education in the UAE (HI, RB, TH) and a research associate (LOA). HI and TH are internal medicine physicians with formal training in medical education; TH has advanced training in PC. RB is an emergency medicine physician. All three physicians completed residency training in Western countries (US- HI, RB, Canada- TH) and served as PDs in UAE residency programs. To minimize bias, we were blinded to participant identities during data analysis. We were mindful of how our academic backgrounds and experiences influenced our analysis of the data and engaged in frequent group conversations to discuss and challenge each other’s interpretations.

## Results

All of the PDs from the seven EM residency programs in the UAE participated in this study. Table 1 lists program demographics. All PDs acknowledged that EM residents routinely care for patients and families who would benefit from PC services and agreed that training in PC is essential in EM residency programs. One PD explained:

I think end-of-life care and palliative care in emergency departments are more common than people think. And I think that’s the mindset that I’d be really glad to see change. [PD4]

**Fig. 1:**
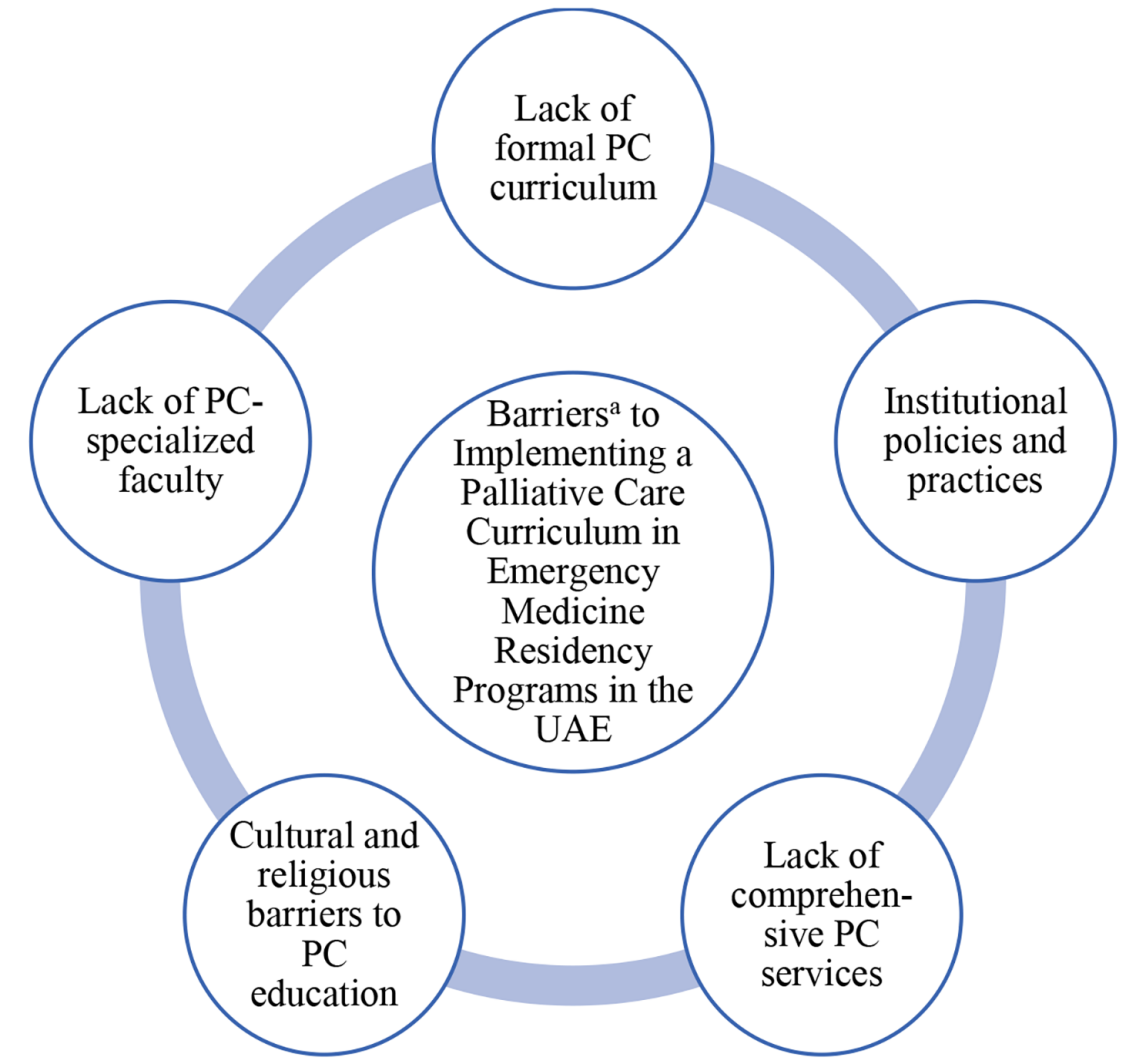
Barriers to implementing a palliative care curriculum in emergency medicine residency programs in the United Arab Emirates. ^a^ as identified by UAE Emergency Medicine Residency Program Directors. PC, palliative care; UAE, United Arab Emirates

Six themes emerged from the interviews, namely the educational curriculum, PC policies and practices, comprehensive palliative care services, cultural and religious barriers to PC, EM scope of practice, and supporting residents after patient death. The themes are discussed below with quotes from the PDs to evidence our findings. The barriers to delivering PC care in EM are summarized in Fig. 1.

**Table 1:** Characteristics of Emergency Medicine Residency Programs in the United Arab Emirates

Emirate	Sponsoring Hospital	Type of Hospital	Palliative Care Services Available	Palliative Care Faculty on staff	Year of Residency Program Development	Total number of residents	ACGME-I Accreditation
Abu Dhabi	Sheikh Khalifa Medical City	Government	No	No	2014	15	Yes
	Cleveland Clinic Abu Dhabi	Private	No	No	2020	8	Yes
	Sheikh Shakhboot Medical City	Government	No	No	2014 (previously Mafraq Hospital)	15	Yes
	Zayed Military Hospital	Military	No	No	2014	12	Yes
	Tawam Hospital	Government	Yes	No	2009	32	Yes
Dubai	Rashid Hospital	Government	No	No	2007	42	No
Sharjah	AlQassimi Hospital	Government	No	No	2019	11	No

ACGME-I, Accreditation Council for Graduate Medical Education International

### Theme 1. Educational curriculum

Despite recognition of its importance, PC education was not a formal component of the EM curriculum in any of the programs and none offered a mandatory rotation in PC. Instead, PC topics were primarily integrated into educational sessions on oncologic emergencies and pain management. Content was often delivered by lecture and case-based learning during academic days. Simulation sessions focused on acute care management topics, with infrequent coverage of serious illness conversations or death notifications. The programs do not routinely integrate other professions, such as nurses, social workers, or faith-based leaders, into the teaching. None of the PDs reported regular assessment of PC knowledge or skills. The PDs explained:

It’s not a mandatory part of our syllabus or in our curriculum. I think it’s more prevalent in the North American curriculum more than the ones in the region. So, in our Arab Board, it’s not there; it’s not mentioned either as a topic to be discussed. But we are sometimes discussing it didactically through lectures. We’ll go through the related chapters in our books and then we’ll speak about it. We are also doing some case-based discussions. But is it a structured one? I will say no. Do we cover PC in our academic day? Yes, we have it as one or two sessions related to the chapters in the book. [PD 2]

There’s no formalized curriculum for PC and that’s not on our academic days. We haven’t done anything like that. We realized there were gaps in being comfortable using opiates and certainly high-dose opiates. But it wasn’t necessarily a focus for end-of-life care- it was just in general. [PD 3]

I do believe it’s probably part of the curriculum, but I don’t think it has significant weight, which is kind of ironic because my background of training was from the UK and, obviously, we have a very different demographic but PC and end-of-life decision making are very much more prominent back in the UK than I’ve noticed here. And it is certainly something that I feel is important for our residents to be trained on. [PD 4]

### Theme 2. PC policies and practices

The PDs reported that resident education is impacted by institutional policies and practices related to death and dying. Several PDs cited the lack of awareness of hospital policies regarding non-escalation of care to be a barrier to the implementation of PC education. Others noted the logistics of initiating do-not-resuscitate (DNR) orders, which require the agreement and signatures of three consultant physicians. The PDs explained:

Until recently, we didn’t have DNR orders in place. It’s only maybe over the last 12 to 14 months, maybe a bit longer, that we’ve been allowed to do it. So, quite often, when somebody comes with probably end-of-life care, we would speak to the critical care consultant and he would initiate the DNR order… and then we’ll put a plan in place. And then if pain relief is required, pain relief will be given in the ER and then he will go to a ward. And then once he goes to the ward and the palliative team will pick him up and whether he needs something for the secretions or whatever, all of that will be taken care of. [PD 1]

I think the barrier often is the logistics of going to sign a DNR. If I’m on a night shift at 3 o’clock in the morning, do I physically get two other consultants to sign the form? It’s impossible. So then you’re in this weird sort of situation where the admitting medical team and the specialists and the juniors from the medical team completely agree with the emergency team about it. But legally we can’t do it because we can’t get the signatures. [PD 4]

### Theme 3: Comprehensive palliative care services

The PDs reported that the lack of a multidisciplinary PC team in many hospitals impacted the ability to formalize PC training and experience for EM residents. One PD elaborated:

We don’t have a structured block. Part of it is because everything has been so fragmented. Even now I’m not saying we have a fantastic PC service or anything. There is still a need to develop and grow. But we have bits and pieces of the element. We have a pain team. We have another social care team, but kind of if you ask me, are they all working in sync together and smoothly? We are not quite there yet. [PD 1]

The lack of community PC services also hindered the EM physician’s ability to care for patients with PC needs. One PD highlighted:

Emergency departments have always been the sort of safety net where patients with PC needs end up and when families are struggling. So, we often see patients where the community setting hasn’t really been able to provide the care that they’ve required and as a result, they’ve ended up in the emergency room. [PD 4]

Another PD added:

In the UK, if someone has terminal cancer, there’s a lot of support. There are specialist nurses who go home to review them and there’s a good primary care setup. So, those nurses and GPs will go check on these patients and make sure their pain is controlled and they die with dignity at home. We don’t have those yet in the UAE. So those are a bit of a challenge. [PD 6]

### Theme 4. Cultural and religious barriers to PC

Most of the PDs felt that the culture and religion of the Middle East were a challenge to initiating PC communication and services. Patient awareness and acceptance of PC were concerns. One PD discussed how escalation of care can be initiated due to family pressures:

You can see a lot of physicians are often under a lot of pressure from families to do things that you probably think that the clinician doesn’t feel very comfortable about, but we’re not really in a position where we can do otherwise. [PD 1]

More importantly, the PDs felt that cultural and religious objections of the healthcare team were barriers to providing effective palliative and end-of-life care. One PD described the reluctance of the healthcare team to initiate PC services:

And the last two years, I think with the implementation of the organ transplant and DNR policy in the UAE, we were talking a little bit more about end-of-life care. But until now, it did not reach the residents because we are teaching, or we are educating, the trainers themselves- the faculty and staff about these concepts. Till now, we have reluctance related again to the cultural background of the emergency department team. Many doctors will say “no, it [DNR] is haram [a sin]. I cannot take the decision to stop this patient from the ventilator or this one. Or we cannot discuss this issue till now.” There are some taboos in this region. [PD 2]

### Theme 5. EM scope of practice

Only one of the PDs felt that PC was not part of the ED’s physician’s mission and should be deferred to other specialties:

I think the right person to have end-of-life conversations would be the specialist, like the oncologist, because the emergency physician might not have all the information and also, they might not be the right person to be put into the situation. The family keep asking questions outside of the emergency physician’s expertise. [PD 6]

Overall, the PDs acknowledged the importance of initiating goals-of-care conversations and educating their trainees to do so:

There’s that I want to do everything because I can rather than it is actually the right thing to do. And I think that’s something certainly we’ve had conversations with our residents and we discussed cases during teaching where we bring these sort of matters up and say you know, it is important from a patient experience for the emergency department to set the right tone. Then that becomes easy for the admitting specialties. And I think that goes a long way to that patient having a good outcome. [PD 4]

Another PD concurred:

Making resuscitative decisions was very difficult. But now in the last two years, we have advanced in this respect and discussing with the families and involving our junior residents most of the time. For example, for people who would not get benefit out of intensive care unit (ICU) treatment and the care is gonna be futile or somebody is post-cardiac arrest or is a sick 90-year-old person. When to make a decision not to do further resuscitation and discuss with family about futility of care- so that’s been part of our teaching program. [PD 5]

### Theme 6. Supporting residents after patient death

The PDs all admitted that patient death in the ED was a source of distress for their trainees. Although most programs lacked formal processes for psychological support, many mechanisms were in place to help trainees cope with the emotions inherent in dealing with death and dying. The most common support was a “hot debrief,” which often occurred within hours of the event. Debriefs focused primarily on clinical reviews of the case but also allowed participants to express emotions and receive support. Other mechanisms included a well-being curriculum with topics on self-care, peer mentoring, an open-door policy of PD and core faculty for informal discussions and support, and reflective practice. The PDs described the support mechanisms available:

Yes, but it’s not a formalized process. I mean, when we have trainees that have had these situations happen, it gets escalated to somebody in our core faculty, if they weren’t already involved. And then we individually reach out to them [the resident] and follow through, but not in a formalized process… But we don’t have a mechanism for formally addressing that outside of a debrief. It’s just, we heard XY and Z happened with a certain resident. Let’s follow up with them rather than some sort of mechanism or trigger to capture all of those cases. [PD 3]

As part of the residency, we’re pushing the notion of reflective practice as well. So, as part of our case-based discussions, the individual that may present will certainly try and encourage them to reflect on it as well….So yeah, it’s something we’re trying to embed as a process to become used to doing because it is a challenge… We also set aside time during our teaching every week. We talk about resilience, we talk about coping strategies. We talk about, you know, how people are feeling. So yeah, we’re very much in tune with that and I think we’re better at that post-code as well. [PD 4]

Within that shift, we’ll do a de-brief. We’ll talk about it. We’ll make sure they understand that they’ve done everything they need to do, and if they need to be released for the day, they can go and then I will follow up with them as a program director. [PD 7]

## Discussion

This study provides a snapshot of PC education in EM residency training programs in the UAE. The study adds to the literature by providing the perceptions and intentions of EM PDs and identifying barriers they faced in providing PC education. Overall, EM PDs agreed that proficiency in palliative and end-of-life care was essential but the lack of a formal curriculum, inconsistencies in the awareness and implementation of institutional policies, the lack of comprehensive and multidisciplinary PC services, and cultural and religious factors served as barriers to initiating PC services and teaching PC competencies in the ED. Our findings are consistent with studies in multiple countries worldwide reporting insufficient PC training in EM residency curricula [24, 25]. In a survey of over 100 EM PDs in the US, just over half of the programs included PC training [26]. A national study of the Canadian College of Family Physicians Emergency Medicine (CCFP(EM)) and the Royal College of Physicians and Surgeons of Canada Emergency Medicine (RCPSC-EM) postgraduate training programs showed that only 38.5% of responding programs had a structured curriculum in palliative and end-of-life care and all education was lecture or seminar-based [27].

The PDs in our study all planned to implement additional PC training sessions. Studies show that PC education should not be limited to didactics. Effective educational modalities include bedside teaching, small group sessions, role-playing, and simulation [28–30]. Research reveals that EM residents desire further training in PC and this training improves comfort and confidence in managing end-of-life patients [14]. For example, EM clinicians in Australia who participated in PC training felt comfortable managing people with advanced cancer presenting to the ED [31]. Even brief interventions have been shown to improve PC attitudes and skills. In one study, a 4-hour educational session improved EM resident comfort in discussing end-of-life care and knowledge of PC concepts, which was maintained at 6 months [14].

Based on our findings, we believe that a structured PC curriculum, with both didactic and clinical components, should be a mandatory part of all EM residency programs in the UAE. Research supports longitudinal teaching throughout the continuum of medical training as the most effective way to improve trainee PC knowledge and skills [32]. Given the strong interest in incorporating PC education despite the lack of structured curricula, we believe that a national teaching framework for culturally competent and locally relevant PC should be adopted by the nation’s EM residency programs to standardize exposure and learning. This will require the recruitment of multidisciplinary PC specialists, including PC nurses, social workers, and faith-based professionals, who can develop and deliver the curriculum and role model the care. Studies show that EOL teaching by PC specialists improves trainee self-efficacy in PC [33]. Faculty development is also necessary. Training programs in goals-of-care communication skills, pain management, and end-of-life symptom management should be available for EM healthcare professionals at all levels to improve their knowledge and skills of core PC principles.

Our findings also have policy implications. Table 2 shows the implications for curricular and policy reform. We are encouraged that several of the PDs routinely initiated PC communication and care within the ED. The ED sets the stage for future inpatient care and determines disposition to the intensive care unit. Early goals-of-care discussions can help tailor treatment plans that are concordant with patient and family preferences. However, initiating end-of-life conversations can be daunting for physicians. Several studies have shown the utility of conversation guides to facilitate clinician-led advance care planning and other electronic resources that are compatible with hospital electronic medical records to support shared decision-making [34]. These resources can be implemented in UAE EDs to facilitate the early provision of PC services.

**Table 2:** Implications for Curricular and Policy Reform

Pertinent Findings from the Study	Implications for Curricular and Policy Reform
EM residency programs do not have a formal palliative care curricula.	Formal palliative care curricula should be developed for EM residency programs and should cover the core PC and EOL competencies for EM physicians. The HPM-EM domains developed by Shoenberger et al.^18^ can be adopted to create a culturally and locally relevant PC curriculum for UAE’s EM programs.
Hospitals lack comprehensive PC services.	Hospitals should recruit multidisciplinary PC specialists, including PC physicians, nurses, social workers, and faith-based professionals who can provide in-hospital PC care and link with community resources. The multidisciplinary team can role model and teach effective PC skills. Professional development programs in PC principles, goals of care communication skills, and pain and EOL symptom management should be available for all EM healthcare professionals.
There is a cultural reluctance to adopt end-of-life care policies.	Nation and hospital-wide educational campaigns should take place to raise awareness on DNR policies. The Emirates Palliative and Supportive Care working group can provide guidance and support in developing culturally acceptable EOL communication and symptom management guidelines.
EM residency programs have many mechanisms to support residents after patient death but lack a formalized process.	EM programs should integrate other professions, including social workers, psychologists, and faith-based leaders, in bereavement debriefing to address resident emotions and psychological well-being after patient death. Programs should also offer debriefing workshops to faculty and residents to build their skills providing resident and peer support after distressing patient care events.

EM, Emergency Medicine; PC, palliative care; EOL, End-of-life; HPM, Hospice and Palliative Medicine; DNR, Do not resuscitate.

Some of the ED clinicians had misconceptions about PC and cultural and religious objections to initiating PC services. Prevailing medical teacher beliefs and cultural views about PC can negatively impact resident education [35]. To fully integrate PC into EDs in the UAE, awareness campaigns on UAE policies and laws regarding DNR are needed to clarify confusion and better inform physicians. Train-the-trainer sessions focusing on the benefits of early PC referrals and the use of opiates in end-of-life pain management can further promote the implementation of PC in the country’s EDs.

A notable strength of the UAE EM residency programs is the focus on resident well-being and support. Residents in programs worldwide are uncomfortable and feel ill-prepared to deal with dying patients and their families [36–38]. Moreover, residents can experience significant grief after a patient’s death [37, 39]. Most of the programs conducted “hot debriefs” shortly after a death in the ED. Studies show that real-time debriefing and supportive discussions can be effective in addressing resident emotions after a patient’s death [40]. The programs also offered peer mentorship and faculty support. Support is an important resource in reducing moral distress and burnout in healthcare professionals [41]. Workshops in peer debriefing should be considered as an additional mechanism to provide trainees with the skills and tools to support their colleagues in the immediate aftermath of a patient’s death [42].

### Limitations

Our study has several limitations. Although a small number of PDs were interviewed, they represented all of the accredited EM residency programs in the UAE at the time of the study. We believe our findings represent the current status of EM PC education. The PDs may have presented their programs in a favorable light, thereby, overestimating the depth and breadth of PC training. We only report the presence of PC training but are unable to assess the quality of the education. Finally, the resident, patient, and family perspectives are missing. Future studies are needed to assess the impact of PC training on EM residents and their competence and skills in palliative and end-of-life care.

## Conclusion

A PC approach provides patients and their families an improved quality of life throughout the duration of an illness and at the end of life [3] and should be provided to hospitalized patients dealing with serious illnesses by all health professionals. There is consensus that proficiency in palliative and end-of-life care is essential but remains a global challenge for physicians. EM residents are a particularly critical group when it comes to PC training. UAE national EM residency curriculum development continues to evolve with major emphases on improving serious illness communication skills and the development of structured PC curricula. As EM programs implement policies and programs to improve communication and care for patients and families dealing with serious illness, future studies are needed to assess the impact of these initiatives on patient quality of life and physician well-being.

### Electronic supplementary material

Below is the link to the electronic supplementary material.

Supplementary Material 1

Supplementary Material 2: Appendix E1. Interview Guide.

## Data Availability

No datasets were generated or analysed during the current study.

## Abbreviations

EM: Emergency medicine
UAE: United Arab Emirates
PC: Palliative care
ED: Emergency departments
PD: Program Directors
ACEP: American College of Emergency Medicine
