# Supplementary material for: Palliative care education: a nationwide qualitative study of emergency medicine residency program directors in the United Arab Emirates
Source: Int J Emerg Med. 2024 May 23;17:69. doi: 10.1186/s12245-024-00643-z (PMC11119274; doi:10.1186/s12245-024-00643-z)
Supplement: Supplementary file 1 — Supplementary Material 1 [file 12245_2024_643_MOESM1_ESM.docx]

**APPENDIX E1**

Interview guide

**Program Demographics:**

What year was the program established?

How many residents are currently in the program?

Is your program accredited by any national/international accreditation bodies?

**Palliative Care Teaching and Resources**

Do you have a formal, structured PC or EOL curriculum?

Why? Why not?

Who/what led to the introduction of the teaching?

If yes, what topics are covered in the curriculum?

If not, are your residents given any formal teaching on PC or EOL care?

Do you have any topics that are related to palliative care but covered under different lectures for example geriatrics, anesthesia, ethics, or communication skills? Can you give me examples?

Are there separate sessions for pediatric and adult palliative care?

What format do you use for palliative care education for your residents?

Does your program have any faculty members who are trained in palliative medicine?

Are any of the following topics covered?

**Domain 1: provider skill set (primary level)**

Pain control

Chronic pain

Malignant and nonmalignant pain

Treating other distressing symptoms (e.g., nausea/vomiting, dyspnea)

Care for the imminently dying (expected death within hours to days)

Non-initiation of non-beneficial interventions

End-of-life management in a mass casualty incident/event

Family witnessed resuscitation

Difficult communication

Delivery of bad news (e.g., prognosis and death telling)

Conflict resolution (e.g., between family members)

Goals of care discussions

Assisting families with decision-making

Assisting patients with decision-making

Caregiver support

Bereavement and grieving

**Domain 2: recognition of PC needs in ED patients**

Trajectories of dying

(Terminal illness, Organ failure, Frailty, Sudden death)

Prognostication

Screening for palliative care needs

Identifying patients who may benefit from PC referral

Identifying imminently dying patient (expected death hours–days)

Rapid palliative care assessment

Aligning diagnostics and therapeutics to patient goals

Functional, psychosocial, and spiritual assessment

Assessing for and initiating hospice referrals

Toolkits to help identify patient for appropriate referrals/resources

Caregiver burden

Complications of cancer

Disease complications (spinal cord compression, hypercalcemia)

Treatment complications (pancreatitis, tumor lysis, AKI)

Spiritual, and cultural issues around EOL and death

**Domain 3: logistic understanding related to PC in the ED**

Allow natural death

Ethical and legal issues

Decision-making capacity

Futility

**Palliative Care experience**

Are your residents involved in taking care of terminally ill patients or patients at EOL?

In what clinical settings does palliative care teaching take place at your institution?

How much exposure do you think your residents have to such patients?

Does your program offer a mandatory or elective PC rotation?

**Coping and self-care**

Are resident coping and self-care part of the curriculum?

Is there a formal debrief after patient deaths? (who runs it?) When does it take place?

Do residents have a mechanism to help cope with patient death?

How do you think your residents are impacted by patient death?

**Evaluation**

Do you evaluate your residents’ knowledge in palliative care? How?

Do you think your residents are competent to manage patients and families with PC/EOL care needs?

**Learning Needs and barriers**

Is it important to your institution that residents learn about how to provide care for dying patients?

Why?

Would you be supportive of integrating end-of-life care into your curriculum?

Why?

How would you integrate it?

In your opinion, what are potential barriers to teaching end-of-life care in EM residency training?
